# Author Correction: Natural reversion promotes LPS elongation in an attenuated *Coxiella burnetii* strain

**DOI:** 10.1038/s41467-024-45637-w

**Published:** 2024-02-12

**Authors:** Carrie M. Long, Paul A. Beare, Diane Cockrell, Picabo Binette, Mahelat Tesfamariam, Crystal Richards, Matthew Anderson, Jessica McCormick-Ell, Megan Brose, Rebecca Anderson, Anders Omsland, Talima Pearson, Robert A. Heinzen

**Affiliations:** 1 Laboratory of Bacteriology, Division of Intramural Research, National Institute of Allergy and Infectious Disease, National Institutes of Health, Hamilton, MT 59840 USA; 2 Office of the Director, Office of Research Services, Division of Occupational Health and Safety, National Institutes of Health, Bethesda, MD 20892 USA; 3 Office of the Director, Office of Research Services, Division of Occupational Health and Safety, National Institutes of Health, Hamilton, MT 59840 USA; 4 Paul G. Allen School for Global Health, College of Veterinary Medicine, Washington State University, Pullman, WA 99164 USA; 5 Department of Biological Sciences, Center for Microbial Genetics and Genomics, Northern Arizona University, Flagstaff, AZ 86011 USA

**Keywords:** Bacterial pathogenesis, Bacteriology, Pathogens

Correction to: *Nature Communications* 10.1038/s41467-023-43972-y, published online 24 January 2024

In this article, the affiliation details for Megan Brose, Rebecca Anderson were incorrectly given as ‘Paul G. Allen School for Global Health, College of Veterinary Medicine, Washington State University; Pullman, 99164, USA’ but should have been ‘Office of the Director, Office of Research Services, Division of Occupational Health and Safety, National Institutes of Health; Hamilton, 59840, USA’.

The original article has been corrected.

