# Supplementary material for: Natural reversion promotes LPS elongation in an attenuated Coxiella burnetii strain
Source: Nat Commun. 2024 Jan 24;15:697. doi: 10.1038/s41467-023-43972-y (PMC10808227; doi:10.1038/s41467-023-43972-y)
Supplement: Supplementary file 1 — Supplementary Information [file 41467_2023_43972_MOESM1_ESM.pdf]

Supplementary Materials for

Natural reversion promotes LPS elongation in an attenuated *Coxiella burnetii* strain

Carrie M. Long, Paul A. Beare, Diane Cockrell, Picabo Binette, Mahelat Tesfamariam, Crystal Richards, Matthew Anderson, Jessica McCormick-Ell, Megan Brose, Rebecca Anderson, Anders Omsland, Talima Pearson, Robert A. Heinzen

Corresponding author: carrie.long@nih.gov

The PDF file includes:

Figs. S1 to S5
Tables S1 to S3

A

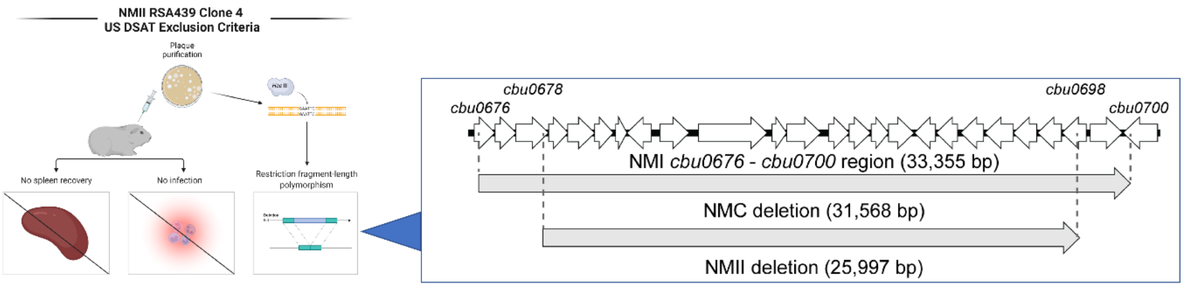

Fig. S1. - *Coxiella burnetii* LPS structure is impacted by genetic factors and associated with NMII RSA439 strain DSAT select agent exemption

(**A**) DSAT select agent exemption criteria for *C. burnetii* NMII RSA439, clone 4. Outcomes following guinea pig intraperitoneal infection and genetic analysis are visually described. Genetic deletions in LPS-related genes (*cbu0676*-*cbu700*) exhibited by NMC and NMII compared to NMI are shown. This figure was created with BioRender.com.

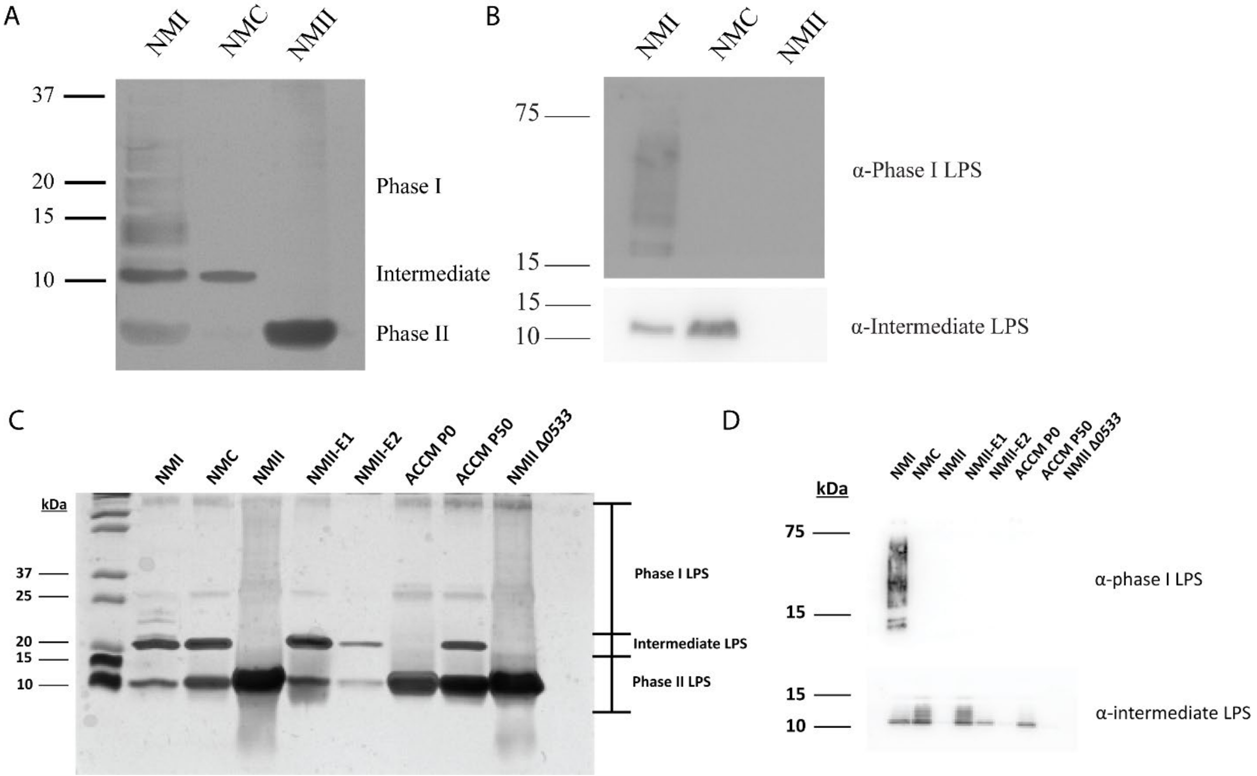

Fig. S2.

LPS profiles of guinea pig infection inocula. Initial inocula following silver stain (**A**) and immunoblot (B). Secondary study inocula following silver stain (C) and immunoblot (D). Data are the result of one independent experiment. Source data are provided as a source data file.

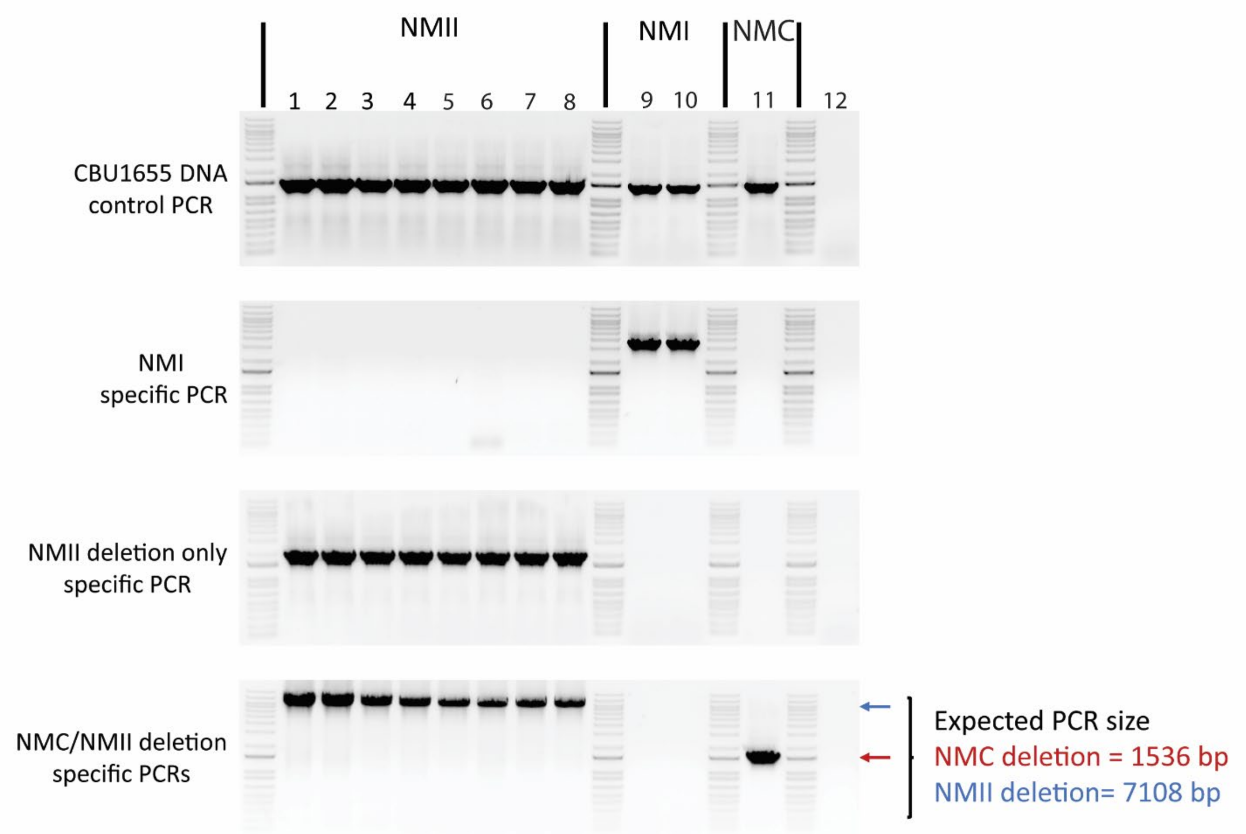

Fig. S3 Genetic analysis of NMC and NMII deletions

Genomic DNA isolated from NMI, NMC, and NMII strains were used as template DNA and PCR was performed using PCR primers to detect (**A**) the presence of *C. burnetii* DNA using PCR primers specific to cbu1655, (B) the NMI genome only, (C) the presence of the large NMII-specific ~26 kb deletion or (D) the presence of either the NMC (~32 kb deletion) or NMII. The 1 kb plus DNA marker was used for sizing. Data are the result of one independent experiment. Source data are provided as a source data file. The location of the primers is outlined in Fig. S4.

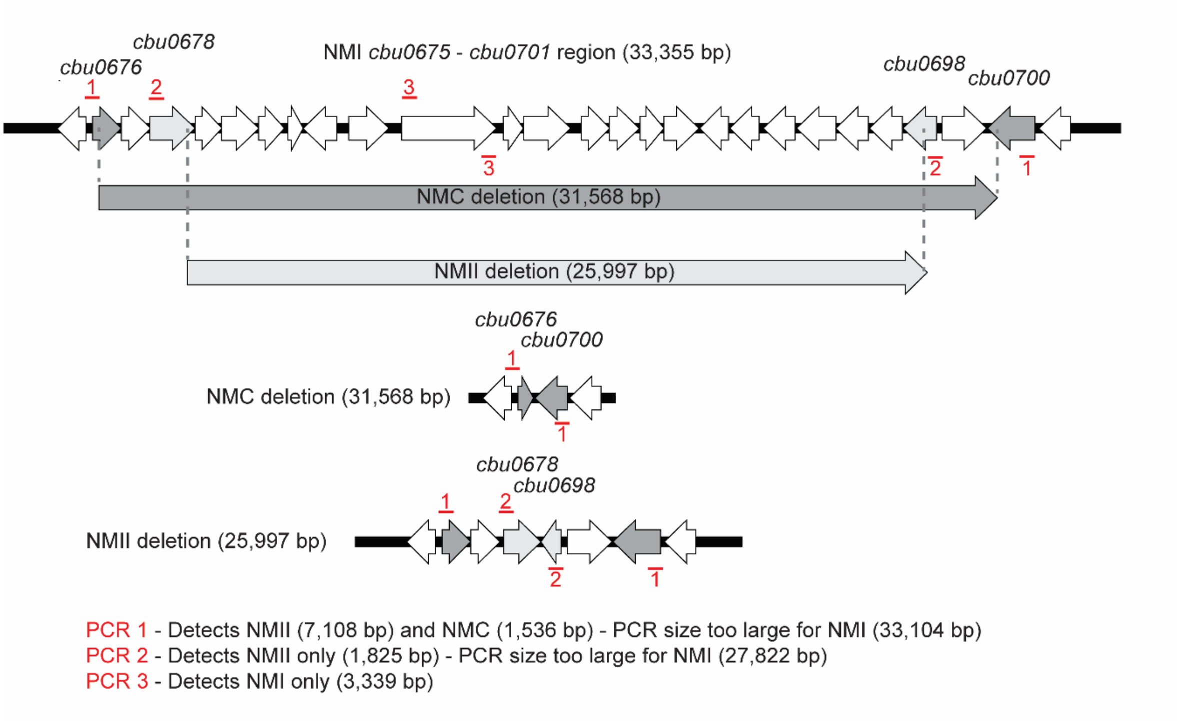

Fig. S4 Key for genetic analysis of NMC and NMII deletions
The figure outlines the location of the PCR primers used in Fig. S3.

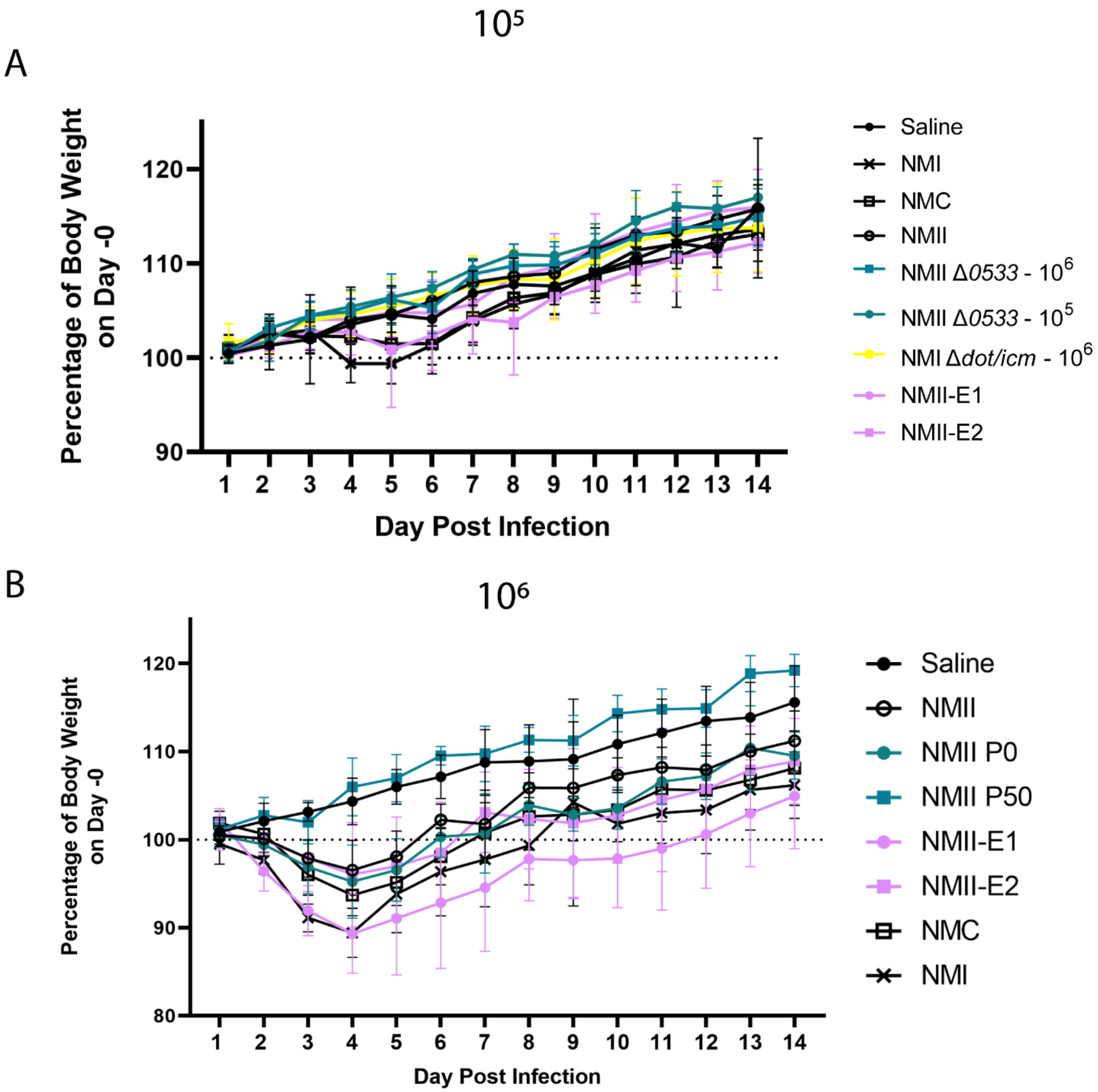

Fig. S5. Post-inoculation guinea pig body weight change kinetics

Post-inoculation guinea pig body weight change is shown following 10^5 (**A**) and 10^6 (B) GE infections. For all graphs, horizontal bars indicate the group means and error bars represent the group standard deviation. Animal group n=6. Data are the result of one independent experiment per graph. Source data are provided as a source data file.

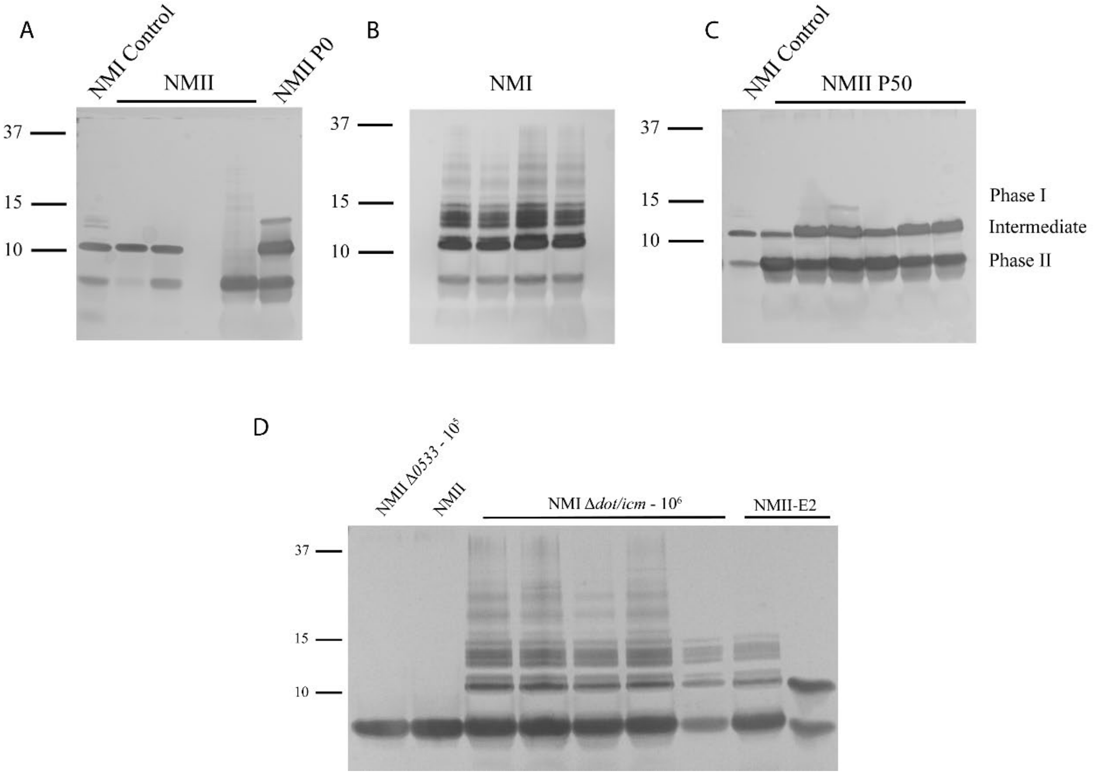

Fig. S6. Post-infection guinea pig spleen LPS profiles

LPS profiles of splenic-derived ACCM-2 outgrowth following infection with 10^6 GE of NMII and NMII P0 (**A**), NMI (B), NMII P50 (C), and 10^5 GE of NMII Δ*0533*, NMII, and NMII-E2 strains and 10^6 GE of NMI Δ*dot/icm* (D). Data are the result of one independent experiment per dosing range. Source data are provided as a source data file.

Group	**Infection Strain**	**Dose (GE)**	**Animal ID**	**# Grown**
1	Saline	-	1-6	0
2	NMII Δ*0533*	10^5	7-12	0
3	NMII Δ*0533*	10^6	13-18	1
4	NMII	10^5	19-24	1
5	NMI Δ*dot/icm*	10^5	25-30	5
6	NMII-E1	10^5	31-36	0
7	NMII-E2	10^5	37-42	2
8	NMC	10^5	43-48	1
9	NMI	10^5	49-54	4
Group	**Infection Strain**	**Dose (GE)**	**Animal ID**	**# Grown**
1	Saline	-	1-6	0
2	NMII	10^6	7-12	4
3	NMII P0	10^6	13-18	1
4	NMII P50	10^6	19-24	6
5	NMII-E1	10^6	25-30	4
6	NMII-E2	10^6	31-36	4
7	NMC	10^6	37-42	2
8	NMI	10^6	43-48	4

Table S1.

Splenic *C. burnetii* outgrowth following infection and axenic culture. Data are the result two independent experiments, as noted in the table.

	Mutations in *cbu0113*					
**Genome**	**Mutation**	**Mutation percentage**	**Coverage**	**Mutation**	**Mutation percentage**	**Coverage**
**NMII**	ins A - FS	0.2%	408	-	-	-
**NMII Δ*cbu0533***	G-A - Stop	100.0%	316	-	-	-
**NMC**	-	-	-	-	-	-
**NMII P2 ACCM-1**	Del T - FS	16.9%	71	Ins A - FS	22.9%	57
**NMII P26 ACCM-1**	Ins C - FS	25.0%	20	G-A - Stop	17.4%	46
**NMII P51 ACCM-1**	Del T - FS	87.0%	23	-	-	-
**NMII-E1**	-	-	-	-	-	-
**NMII-E2**	-	-	-	-	-	-

Table S2.
Specific *cbu0113* mutation coverage in various *C. burnetii* strains following LPS elongation.

NCBI BioSample Accession	Identification ID	
SAMN38068166	NMII RSA439 Passage 2	https://www.ncbi.nlm.nih.gov/biosample/SAMN38068166/
SAMN38068167	NMII RSA439 Passage 26	https://www.ncbi.nlm.nih.gov/biosample/?term=SAMN38068167
SAMN38068168	NMII RSA439 Passage 51	https://www.ncbi.nlm.nih.gov/biosample/?term=SAMN38068168
SAMN38068169	NMC	https://www.ncbi.nlm.nih.gov/biosample/?term=SAMN38068169
SAMN38068170	NMII RSA439	https://www.ncbi.nlm.nih.gov/biosample/?term=SAMN38068170
SAMN38068171	NMII RSA439 Δ*cbu0335*	https://www.ncbi.nlm.nih.gov/biosample/?term=SAMN38068171
SAMN38068172	NMII RSA439 E1	https://www.ncbi.nlm.nih.gov/biosample/?term=SAMN38068172
SAMN38068173	NMII RSA439 E2	https://www.ncbi.nlm.nih.gov/biosample/?term=SAMN38068173
SAMN38068174	NMII RSA439 GP	https://www.ncbi.nlm.nih.gov/biosample/?term=SAMN38068174

Oligonucleotide	
PCR primers for analysis of large chromosomal deletions	Sequence 5'-3'
CBU1655-F	
CBU1655-R	GTGGCATAATAAGCGCTCGTTTC
NMIonly-det-F	CGATTTGGCTGGGTTGGTAATCGC
NMIonly-det-R	ATGTCTAAACCACTCGCTATTATAAAG
NMIIonly-deldet-F	TCAACTAAAATGATTTTTTGGCTCGG
NMIIonly-deldet-R	ATGTTGCTGAAACGATACCGTC
NMC/NMII-deldet-F	ATGATACGCCCTAAGAGAGTTTG
NMC/NMII-deldet-R	ATGACCAAACGATTTGATCGTATATTAG
	ATGCGTTCATTGATTTTGAACCCG
PCR primers for cbu0335 deletion	
CBU0335-KO-5'F	
CBU0335-KO-5'R	CGGTACCCGGGGATCCGGATTAAGCTCG AAATCATCGGC
CBU0335-KO-3'F	CACCCATATGCGACGCGAGCGTCGAGTA AGATCCTCCCACAAGTTAATCAAATC
CBU0335-KO-3'R	CGTCGCATATGGGTGCGCATGTACGTCA TTCCCTGAGGTGCGAGAGTCGTTC
	GAACCTGTTTGTCGACCCTCTTGAGCTT GGAAATTCCGATG
PCR primers for cbu0533 deletion	
CBU0533-KO-5'F	
CBU0533-KO-5'R	CGGTACCCGGGGATCCCAGACCCAAAA GTTATTGTGGC
CBU0533-KO-3'F	CACCCATATGCGACGCGAGCGTCGAGA AAAAGTCACATCCTGCAGTTCT
CBU0533-KO-3'R	CGTCGCATATGGGTGCGCATGTACGTCG CTCTTGTGACTAAAACCTCC
	GAACCTGTTTGTCGACTTCCAGCAAAGG ATCGAACTGG

Table S3.

Oligonucleotide primer sequences utilized in this study.
