# Supplementary material for: Natural reversion promotes LPS elongation in an attenuated Coxiella burnetii strain
Source: Nat Commun. 2024 Jan 24;15:697. doi: 10.1038/s41467-023-43972-y (PMC10808227; doi:10.1038/s41467-023-43972-y)
Supplement: Supplementary file 3 — Reporting Summary [file 41467_2023_43972_MOESM3_ESM.pdf]

nature portfolio

Corresponding author(s): Carrie Long

Last updated by author(s): 2023-10-5

Reporting Summary

Nature Portfolio wishes to improve the reproducibility of the work that we publish. This form provides structure for consistency and transparency in reporting. For further information on Nature Portfolio policies, see our Editorial Policies and the Editorial Policy Checklist.

Statistics

For all statistical analyses, confirm that the following items are present in the figure legend, table legend, main text, or Methods section.

n/a	Confirmed	
☐	☒	The exact sample size (*n*) for each experimental group/condition, given as a discrete number and unit of measurement
☐	☒	A statement on whether measurements were taken from distinct samples or whether the same sample was measured repeatedly
☐	☒	The statistical test(s) used AND whether they are one- or two-sided<br>*Only common tests should be described solely by name; describe more complex techniques in the Methods section.*
☐	☒	A description of all covariates tested
☐	☒	A description of any assumptions or corrections, such as tests of normality and adjustment for multiple comparisons
☐	☒	A full description of the statistical parameters including central tendency (e.g. means) or other basic estimates (e.g. regression coefficient) AND variation (e.g. standard deviation) or associated estimates of uncertainty (e.g. confidence intervals)
☐	☒	For null hypothesis testing, the test statistic (e.g. *F*, *t*, *r*) with confidence intervals, effect sizes, degrees of freedom and *P* value noted<br>*Give P values as exact values whenever suitable.*
☒	☐	For Bayesian analysis, information on the choice of priors and Markov chain Monte Carlo settings
☒	☐	For hierarchical and complex designs, identification of the appropriate level for tests and full reporting of outcomes
☒	☐	Estimates of effect sizes (e.g. Cohen's *d*, Pearson's *r*), indicating how they were calculated

Our web collection on statistics for biologists contains articles on many of the points above.

Software and code

Policy information about availability of computer code

Data collection	No data collection programs were used.
Data analysis	The following software programs were used for data analysis and presentation: Adobe Illustrator (Version 28), GraphPad Prism (Version 9.3.1), Geneious (Version 2021.1.1), and Microsoft Office Suite (Version 2302).

For manuscripts utilizing custom algorithms or software that are central to the research but not yet described in published literature, software must be made available to editors and reviewers. We strongly encourage code deposition in a community repository (e.g. GitHub). See the Nature Portfolio guidelines for submitting code & software for further information.

Data

Policy information about availability of data

All manuscripts must include a data availability statement. This statement should provide the following information, where applicable:

- Accession codes, unique identifiers, or web links for publicly available datasets
- A description of any restrictions on data availability
- For clinical datasets or third party data, please ensure that the statement adheres to our policy

A data availability statement has been provided in the manuscript and accession codes can be found in Table S3.

Research involving human participants, their data, or biological material

Policy information about studies with human participants or human data. See also policy information about sex, gender (identity/presentation), and sexual orientation and race, ethnicity and racism.

Reporting on sex and gender	N/A
Reporting on race, ethnicity, or other socially relevant groupings	N/A
Population characteristics	N/A
Recruitment	N/A
Ethics oversight	N/A

Note that full information on the approval of the study protocol must also be provided in the manuscript.

Field-specific reporting

Please select the one below that is the best fit for your research. If you are not sure, read the appropriate sections before making your selection.

☒ Life sciences ☐ Behavioural & social sciences ☐ Ecological, evolutionary & environmental sciences

For a reference copy of the document with all sections, see nature.com/documents/nr-reporting-summary-flat.pdf

Life sciences study design

All studies must disclose on these points even when the disclosure is negative.

Sample size	Sample sizes were determined by power analysis based on prior experiments.
Data exclusions	No data were excluded from analysis.
Replication	This manuscript describes two animal studies serving as replicates for occurrence of the described biological phenomenon, each yielding cohesive results.
Randomization	Animals were randomized by blinded animal caretakers upon initial housing.
Blinding	The investigators were not blinded due to constraints with ABSL-3 work and Select Agent reporting, which requires strict adherence to guidelines and demarcation of animal identity.

Reporting for specific materials, systems and methods

We require information from authors about some types of materials, experimental systems and methods used in many studies. Here, indicate whether each material, system or method listed is relevant to your study. If you are not sure if a list item applies to your research, read the appropriate section before selecting a response.

Materials & experimental systems

n/a	Involved in the study	
☐	☒	Antibodies
☒	☐	Eukaryotic cell lines
☒	☐	Palaeontology and archaeology
☐	☒	Animals and other organisms
☒	☐	Clinical data
☒	☐	Dual use research of concern
☒	☐	Plants

Methods

n/a	Involved in the study	
☒	☐	ChIP-seq
☒	☐	Flow cytometry
☒	☐	MRI-based neuroimaging

Antibodies

Antibodies used	Mouse monoclonal antibodies AAB-COX-MAB (Bei Resources), 1E4 (Fab1E4, a generous gift of Guoguan. Zhang, University of Missouri-Columbia), and A6 (in house hybridoma) [were used to label phase I, intermediate, and phase II LPS molecules, respectively.

Validation	All anitbodies have been validated by the supplier against C. burnetii LPS and additional validation is described by Beare, et al;, doi: 10.1371/journal.ppat.1006922 and Peng, et al.; doi: 10.1128/IAI.01695-14.

Animals and other research organisms

Policy information about studies involving animals; ARRIVE guidelines recommended for reporting animal research, and Sex and Gender in Research

Laboratory animals	Cavia porcellus (Guinea pig), Hartley Strain (Charles River 051), Female, 6-10 weeks of age.
Wild animals	No wild animals were used in the study.
Reporting on sex	Sex based analysis was not performed as only female animals were utilized. Female animals were chosen based on historical studies and to reduce sex-based variations in results.
Field-collected samples	No field collected samples were used in the study.
Ethics oversight	The NIH/NIAID/RML Institutional Animal Care and Use Committee approved this work (ASP: 2021-067-E).

Note that full information on the approval of the study protocol must also be provided in the manuscript.

Plants

Seed stocks	N/A
Novel plant genotypes	N/A
Authentication	N/A
